# Supplementary material for: Transmission delays and frequency detuning can regulate information flow between brain regions
Source: PLoS Comput Biol. 2021 Apr 15;17(4):e1008129. doi: 10.1371/journal.pcbi.1008129 (PMC8049288; doi:10.1371/journal.pcbi.1008129)
Supplement: Attachment — Submitted filename: PCOMPBIOL-D-20-01170_review.pdf [file pcbi.1008129.s001.pdf]

General comments

Thank you for asking me to review the interesting paper entitled, "Transmission delays and frequency detuning can regulate information flow between brain regions." I think this paper studies a very interesting problem, that is, how the frequencies mismatch and the delays affect the transmission of information in neuronal networks.

However, I think there is a point which can lead to confusion. The authors claim they study the role of frequency mismatch. It is true that varying the parameter Δ the make both networks to oscillate at different frequencies. However, as there is bidirectional coupling, in general (as Fig 2 shows) these two networks tend to phase-lock. Therefore, effectively, they are not studying cases in which both networks oscillate at different frequencies. They are considering how the phase difference between populations induced by the phase-locking at different frequencies (plus the delay) affect the transmission of information. I think this important point is not sufficiently emphasized along the manuscript. I will ask the authors to emphasize it (maybe separating fig 2B from fig 2), because one might think that the manuscript studies frequency mismatch implying non-phase locked scenarios.

Therefore work in this manuscript becomes similar to this one, also published in PLoS Computational Biology
https://journals.plos.org/ploscompbiol/article?id=10.1371/journal.pcbi.1007019
However, in the current manuscript authors obtain their results by means of a more realistic network and study very relevant magnitudes as cross-covariance, coherency and dMI.

My overall impression is that this paper needs a major revision improving their readability. I think some of the results are not sufficiently explained and some parts are difficult to follow. Therefore, despite the research and ideas studied in this manuscript are worth publishing, I will ask for major revisions concerning the redaction of the manuscript and some extra computations to base more in detail the claims in the paper.

Comments

- Line 76. 'We numerically solved the equations using the Milshtein algorithm with an integration step dt = 0.01 ms'. Please add a citation.
- Line 88. 'To test the signal transmission we injected slow (5 Hz) and fast (70 Hz) non-periodic signals ...' I would appreciate if authors can motivate why they consider slow and fast cases.
- Line 94. The sentence 'In each experiment, we applied a rectangular pulse of amplitude

I pulse = 0.25 A/cm 2 and width of 2 (ms)' confuses me. In line 85 you state that the standard deviation of the noise is = 0.5 A/cm 2. It confuses me how is possible to determine the effect of an input which is comparable in magnitude to the noise.

- Line 114. Does X_i refer to the activity of a population? If so, can you shortly indicate it at some point on the explanation of Delayed mutual information?
- Line 126. The sentence 'By recalculating the firing rate of the population, we defined the PRC as the difference between the oscillation period of the population without and with the injected pulse.' confuses me. I guess it shall be an equivalent approach, but it surprises me you do not measure the PRC measuring the phase difference between perturbed and unperturbed trajectories.
- Line 144. According to the caption of Fig 1 although you show two cases, you will just use one (the one in Panel D). Therefore, the sentence in line 'For the set of parameters we have chosen (dots in Fig. 1A-B and Table 1), each population exhibits synchronous oscillations in the high-gamma range. Sample spiking activity of the neurons and the population activity of the networks are shown in Fig. 1C' confuses me because I understand from the caption you work with one set of parameters in Fig 1D.
- Line 149. I think you refer to Fig. 2A
- Fig.2 For the sake of clarity, I think the colorbars for the Figs 2C, 2D shall be outside the plot.
- Line 149. As you said, when the two populations are phase-locked, their frequencies are the same. Indeed, as Fig 2B shows, for the region of parameters that you study, there is a predominance of phase-locking. Therefore I think that a careful revision of the manuscript shall be performed, because sometimes the authors use the term 'frequency mismatch' referring to $\Delta \neq 0$. However this does not necessarily imply that the system achieves an state in which both populations oscillate with different frequencies. In fact, for most of the scenarios studied in the paper, both populations oscillate at the same frequency. This has to be emphasized along the manuscript. I would suggest separate Fig 2B from Fig 2.
- Line 169. 'We consider the two widespread neuronal coding schemes, rate and spike-time coding'. Please add a citation or explain a bit in detail for the non-computational reader why you consider the two different types of signals.
- Line 206. If we denote by θ^* the time at which the population 1 fires, then $nPRC = PRC_2(\theta^* + \Delta\theta + \delta)$, where $\Delta\theta$ will be the delay between the populations due to Δ and δ and PRC_2 stands for the PRC of the second population. θ^* will be related to the PRC of the first population. Indeed if I have understood the paper, since both populations are identical, then $PRC_2(\theta) = PRC_1(\Delta = 0, \theta)$. If this is correct, I think you can even

improve your predictions in Fig. 3A by explicitly computing the nPRC. Anyway, I think you can derive an expression for the nPRC which would be very useful for analyzing the results.

- Another thing I consider will strongly help to emphasize, illustrate and better discuss the role of PRCs would be a figure showing a raster plot of one population and its corresponding PRC. That will help to link the phase with the oscillation itself. For example, I do not know to which point corresponds the zero of the oscillation.

- Line 226. Related to my previous point. 'A good agreement is seen between ... ' I am not sure results show a 'good agreement'. There are pairs for which you prediction works and other for which does not work. A bit more of discussion about why in certain cases the prediction works and in which does not would be good. Indeed, the computation of the nPRC following the above formula (provided it is correct) can even lead to a more clear discussion of how Δ and δ shape the nPRC. Indeed a formula for the nPRC would be a nice addition to the Theoretical results.

- Line 235. 'If the receiver population receives the perturbation (after a transmission time δ) in a phase at which the time-derivative of the PRC is non-negligible (within the non-zero part of the blue solid curves in Fig. 3A), then the receiver population detects the perturbation'. I am not sure of this. Maximal and minimal values of the PRC will have zero derivative as well. However I expect them to produce an effect, so that sentence confuses me.

- Line 243. 'It can be seen that the results agree well with those obtained by analyzing the pulse transmission in Fig. 3B, highlighting the importance of PRC analysis'. Please explain this in more detail.

- Fig. 4. Is the caption correct? PRC' corresponds to the derivative of the PRC, right? Furthermore I think that to make more solid the sentence 'This measure predicts the quality of the transmission' authors shall add a third panel to the Figure computing some magnitude (¿dMI?) showing this. Also I think Figures in panel A should be bigger.

- Line 247. 'Here we question if the above results can predict (and be inferred from) the causal relationship between the populations and the direction of the information flow. To this end, we compute the delayed mutual information (see Methods Eq. 4) between the two populations' Which are the above results? I do not see how you relate the previous results (I assume the ones related with PRCs) with the Fig. 2D.

- Line 350. 'Locked state' corresponds to the dashed line in Fig. 6?

- Line 352. 'The results shown in Fig. 7(A) are in excellent agreement with the analytical results'. Please discuss this sentence in more detail.

- About the discussion, I think is a bit short. For example, it surprises me that section 'Role of the frequency detuning and connection delay', in which the authors shall precisely describe their results based on the motivation of the paper, is just 8 lines. I also would like the authors to consider the previous mentioned paper on PLoS Comp. Bio. since it is related to their research.
