# Supplementary material for: Transmission delays and frequency detuning can regulate information flow between brain regions
Source: PLoS Comput Biol. 2021 Apr 15;17(4):e1008129. doi: 10.1371/journal.pcbi.1008129 (PMC8049288; doi:10.1371/journal.pcbi.1008129)
Supplement: Attachment — Submitted filename: revissions.pdf [file pcbi.1008129.s003.pdf]

I thank the authors for revising the manuscript subject to my suggestions. I also thank Reviewer #2 for his/her interesting suggestions. I think the manuscript has significantly improved. I would like to congratulate the authors for their effort. The manuscript is now better structurated and is easier to read. I am happy to report that I can now recommend the manuscript for publication. Regarding the concerns about novelty and significance, since my backgrund is more mathematics than neuroscience, I will support Reviewer's #2 final decission since his/her comments reflect a huge knowledge of this area.
