# Supplementary material for: Transmission delays and frequency detuning can regulate information flow between brain regions
Source: PLoS Comput Biol. 2021 Apr 15;17(4):e1008129. doi: 10.1371/journal.pcbi.1008129 (PMC8049288; doi:10.1371/journal.pcbi.1008129)
Supplement: Attachment — Submitted filename: Aref-rebuttal20Jan2021.docx [file pcbi.1008129.s002.docx]

Dear editor,

We thank you for your time and the reviewers for precise reading the manuscript and for their constructing comments. Please find below our response to the comments raised by the reviewers (in red) and the changes which has been made accordingly. In particular, the changes also indicate the novelty of the current study in light of previous related papers.

Best regards,

Alireza Valizadeh

On the behalf of coauthors

Reviewer #1

**General comments**

Thank you for asking me to review the interesting paper entitled, “Transmission delays and

frequency detuning can regulate information flow between brain regions.” I think this paper

studies a very interesting problem, that is, how the frequencies mismatch and the delays affect

the transmission of information in neuronal networks.

However, I think there is a point which can lead to confusion. The authors claim they study

the role of frequency mismatch. It is true that varying the parameter Δ the make both networks

to oscillate at different frequencies. However, as there is bidirectional coupling, in general (as

Fig 2 shows) these two networks tend to phase-lock. Therefore, effectively, they are not studying cases in which both networks oscillate at different frequencies. They are considering how the phase difference between populations induced by the phase-locking at different frequencies (plus the delay) affect the transmission of information. I think this important point is not sufficiently emphasized along the manuscript. I will ask the authors to emphasize it (maybe separating fig 2B from fig 2), because one might think that the manuscript studies frequency mismatch implying non-phase locked scenarios.

Thanks for rising this important point. Indeed we followed a convention in the literature of coupled oscillators to use the term “frequency mismatch” for the mismatch in the “natural” frequencies of the oscillators—when they are uncoupled. Please see our response to your detailed comments and the changes made to address this point.

Therefore work in this manuscript becomes similar to this one, also published in PLoS

Computational Biology https://journals.plos.org/ploscompbiol/article?id=10.1371/journal.pcbi.1007019

However, in the current manuscript authors obtain their results by means of a more realistic

network and study very relevant magnitudes as cross-covariance, coherency and dMI.

My overall impression is that this paper needs a major revision improving their readability.

I think some of the results are not sufficiently explained and some parts are difficult to follow.

Therefore, despite the research and ideas studied in this manuscript are worth publishing, I will

ask for major revisions concerning the redaction of the manuscript and some extra computations to base more in detail the claims in the paper.

Thanks for the positive assessment of the manuscript. We now highlighted the results of the mentioned manuscript and compared their findings with ours in the discussion section. See below.

**Comments**

• Line 76. ’We numerically solved the equations using the Milshtein algorithm with an

integration step dt = 0.01 ms’. Please add a citation.

The following reference is added.

M. San Miguel and R. Toral, Stochastic Effects in Physical Systems, Instabilities and Nonequilibrium Structures VI, edited by E. Tirapegui, J. Martínez, and R. Tiemann ~Kluwer Academic, Dordrecht, 2000, pp. 35–130. (Page 4, Line 83)

• Line 88. ’To test the signal transmission we injected slow (5 Hz) and fast ( 70 Hz) nonperiodic

signals ...’ I would appreciate if authors can motivate why they consider slow and

fast cases.

We added a statement and a reference (Kumar, Arvind, Stefan Rotter, and Ad Aertsen. *Nature reviews neuroscience* 11.9 (2010): 615-627) to address this point (Line 181). A paragraph in the discussion section also dedicated to explaining this point. Please see also our response to your related comment on line 169, below.

• Line 94. The sentence ’In each experiment, we applied a rectangular pulse of amplitude

I pulse = 0.25A/cm 2 and width of 2 (ms)’ confuses me. In line 85 you state that the

standard deviation of the noise is = 0.5 A/cm 2. It confuses me how is possible to

determine the effect of an input which is comparable in magnitude to the noise.

As the reviewer notes the effect of the pulse to a single neuron can be washed out by a noise of comparable magnitude. However, since we use uncorrelated white noise its effect is considerably reduced when recording the population activity; while the effect of the signal adds up.

Moreover, we used an ensemble average when extracting the results. Each point is the results of averaging about 20 different realizations of the application of the same pulse.

We corrected the statement in the methods’ section of the new version, and indicated that noises are uncorrelated.

• Line 114. Does *Xi* refer to the activity of a population? If so, can you shortly indicate it

at some point on the explanation of Delayed mutual information?

This is now indicated in the text. (Page 6, Line 126)

• Line 126. The sentence ’By recalculating the firing rate of the population, we defined the

PRC as the difference between the oscillation period of the population without and with

the injected pulse.’ confuses me. I guess it shall be an equivalent approach, but it surprises

me you do not measure the PRC measuring the phase difference between perturbed and

unperturbed trajectories.

We used the standard method by calculating the instantaneous period with and without injection of the pulse. This value is proportional to the PRC with a 2 pi/T factor. The text is now changed to clarify this point. (Page 6, Lines 134)

• Line 144. According to the caption of Fig 1 although you show two cases, you will just use

one (the one in Panel D). Therefore, the sentence in line ’For the set of parameters we have

chosen (dots in Fig. 1A-B and Table 1), each population exhibits synchronous oscillations

in the high-gamma range. Sample spiking activity of the neurons and the population

activity of the networks are shown in Fig. 1C’ confuses me because I understand from the

caption you work with one set of parameters in Fig 1D.

Indeed we have shown the parameter space which is spanned to choose a set of parameters for the rest of the manuscript. We have now clearly indicated, in the text and in the caption of Fig. 1, that the parameters of Fig. 1C, bottom panel, are chosen for the rest of simulations.

• Line 149. I think you refer to Fig. 2A

Corrected.

• Fig.2 For the sake of clarity, I think the colorbars for the Figs 2C, 2D shall be outside the

plot.

Done.

• Line 149. As you said, when the two populations are phase-locked, their frequencies are

the same. Indeed, as Fig 2B shows, for the region of parameters that you study, there is a

predominance of phase-locking. Therefore I think that a careful revision of the manuscript

shall be performed, because sometimes the authors use the term ’frequency mismatch’

referring to Δ *̸*= 0. However this does not necessarily imply that the system achieves an

state in which both populations oscillate with different frequencies. In fact, for most of

the scenarios studied in the paper, both populations oscillate at the same frequency. This

has to be emphasized along the manuscript. I would suggest separate Fig 2B from Fig 2.

We used the convention to use frequency mismatch for unequal natural frequencies of the two networks, when uncoupled, due to the different input levels. This mismatch leads to a certain phase difference in the locked state when the oscillators are coupled as the reviewer mentions.

We agree that this point could be misleading for the readers so we clarified this point, by changing “frequency” to “natural frequency” throughout the manuscript when applicable. We also made changes in the introduction and in the results section to clarify this point. We also separated Fig 2B to highlight the role of the phase locking and phase difference.

Moreover, we added a paragraph in the Discussion section directly addressing this point. (Page 17, line, 448)

• Line 169. ’We consider the two widespread neuronal coding schemes, rate and spike-time

coding’. Please add a citation or explain a bit in detail for the non-computational reader

why you consider the two different types of signals.

We added the a citation to the following review paper which addresses these two coding schemes

Kumar, Arvind, Stefan Rotter, and Ad Aertsen. "Spiking activity propagation in neuronal networks: reconciling different perspectives on neural coding." *Nature reviews neuroscience* 11.9 (2010): 615-627.

A paragraph in the discussion section also dedicated to explaining this point. (Page 18, Line 475)

• Line 206. If we denote by *θ∗* the time at which the population 1 fires, then *nPRC* =

*PRC*2(*θ∗* + Δ*θ* + *δ*), where Δ*θ* will be the delay between the populations due to Δ and *δ*

and *PRC*2 stands for the PRC of the second population. *θ∗* will be related to the PRC

of the first population. Indeed if I have understood the paper, since both populations

are identical, then *PRC*2(*θ*) = *PRC*1(Δ = 0*, θ*). If this is correct, I think you can even

improve your predictions in Fig. 3A by explicitly computing the nPRC. Anyway, I think

you can derive an expression for the nPRC which would be very useful for analyzing the

results.

Indeed calculation of the nPRC is a bit tricky. The point is that (as we have shown in the theoretical results) the nPRC is proportional to the (phase) derivative of ${PRC}_{2}$. The reason is that the signal applied at the sender population is so small that it only changes the timing of its next pulse. This change in the timing translates to the second population with a factor proportional to the derivative of its PRC.

We tried to address this point more clearly in the theoretical background section. We have also highlighted the expression for nPRC in Eq. 10 in the new version.

• Another thing I consider will strongly help to emphasize, illustrate and better discuss the

role of PRCs would be a figure showing a raster plot of one population and its corresponding

PRC. That will help to link the phase with the oscillation itself. For example, I do not

know to which point corresponds the zero of the oscillation.

We have demonstrated the method for calculation of PRC including a raster plot in the Fig. 1D of the revised version.

• Line 226. Related to my previous point. ’A good agreement is seen between ... ’ I am not

sure results show a ’good agreement’. There are pairs for which you prediction works and

other for which does not work. A bit more of discussion about why in certain cases the

prediction works and in which does not would be good. Indeed, the computation of the

nPRC following the above formula (provided it is correct) can even lead to a more clear

discussion of how Δ and *δ* shape the nPRC. Indeed a formula for the nPRC would be a

nice addition to the Theoretical results.

We have now highlighted the derived formula for the nPRC in Eq. 10 of the theoretical background section. For a better comparison of the results between theory and simulation, we have shown the peak value of the two blue and green curves in Figs. 4B and C of the revised paper. It can be better seen now that our theoretical approximation fits well for most values of the parameters in the locked region. We have extended the corresponding text in the revised version, to address the possible reasons of the difference between the theory and simulations. (Page 9, line 233)

• Line 235. ’If the receiver population receives the perturbation (after a transmission time

*δ*) in a phase at which the time-derivative of the PRC is non-negligible (within the nonzero

part of the blue solid curves in Fig. 3A), then the receiver population detects the

perturbation’. I am not sure of this. Maximal and minimal values of the PRC will have

zero derivative as well. However I expect them to produce an effect, so that sentence

confuses me.

We agree that this result is counter-intuitive (see our response to the comment on page 11 line 260). So we extended the explanations in this paragraph and in the theoretical background section to clarify this point.

Please note that population 2 receives the pulse(s) from population 1 in the absence of the external signal. In fact, the effect of the (weak) external signal is not to “create” a pulse that transmits from population 1 to population 2, but it just changes the time of the next spikes of the population 1. Then, the population 2 should detect this change in the timing of the incoming spike packet. That is why the difference of the PRC in the two different times determines the effective impact of the signal on the receiver population. In the case of weak signal this difference is proportional to the phase (or time) derivative of the PRC. The numerical results presented in Fig. 7B supports this theoretical argument in case of phase oscillators.

• Line 243. ’It can be seen that the results agree well with those obtained by analyzing

the pulse transmission in Fig. 3B, highlighting the importance of PRC analysis’. Please

explain this in more detail.

We extended the paragraph to explain how by knowing the PRC of the populations, the quality of the signal transmission can be readily derived. (Page 11, line 272)

• Fig. 4. Is the caption correct? PRC’ corresponds to the derivative of the PRC, right?

Furthermore I think that to make more solid the sentence ’This measure predicts the

quality of the transmission’ authors shall add a third panel to the Figure computing some

magnitude (¿dMI?) showing this. Also I think Figures in panel A should be bigger.

We added a new panel to this figure which shows the information transfer in the network in the absence of the signal. The results in this new panel (Fig. 5C in the new version) conforms the results showing the signal transmission. We also retained just one figure of part A for a clear presentation.

• Line 247. ’Here we question if the above results can predict (and be inferred from) the

causal relationship between the populations and the direction of the information flow. To

this end, we compute the delayed mutual information (see Methods Eq. 4) between the

two populations’ Which are the above results? I do not see how you relate the previous

results (I assume the ones related with PRCs) with the Fig. 2D.

Here we meant to show that the measures which show information transfer give the similar results to those with the (linear) measures for transmission based on correlation and PRC. We changed the statement to clarify this point. (Page 11, line 283)

• Line 350. ’Locked state’ corresponds to the dashed line in Fig. 6?

This is now noted.

• Line 352. ’The results shown in Fig. 7(A) are in excellent agreement with the analytical

results’. Please discuss this sentence in more detail.

Here we meant matching between the simulation Fig. 8A and theory Fig. 7C (in the new version). We reworded the text to clarify.

• About the discussion, I think is a bit short. For example, it surprises me that section

’Role of the frequency detuning and connection delay’, in which the authors shall precisely

describe their results based on the motivation of the paper, is just 8 lines. I also would

like the authors to consider the previous mentioned paper on PLoS Comp. Bio. since it is

related to their research.

Thanks for this notice. We extended the Discussion and added several explanations in response to the points raised by the reviewer. We also added the relevant references and discussed the similarities and differences of the current study with previous ones.

Reviewer #2: The authors analyze the impact of transmission delays and frequency detuning for the information flow between brain regions. For this they use 2 populations of Hodgkin Huxley neurons and analyze the coherence, mutual information and the phase response curves. The manuscript is well written and seems technically sound and the results are sound and interesting, but a link to the literature on the impact of time-delays in oscillatory brain network models and neuronal fields is missing.

Thanks for your comments. In the revised version we have tried to more clearly show the relation of our results to previous studies by expanding the discussion and adding new references. Please see below.

Below are my specific comments:

- Neural field dynamics with local and global connectivity and time delay has been studied for more than 10 years, see e.g. Jirsa PTRSA 2009.

Thanks for introducing this important reference. This is now cited in the introduction when effect of delay is discussed. (Page 3, line 50)

- The impact of time-delays for the large scale brain dynamics has also been extensively studied, both analytically and computationally. Authors should make an effort to discuss their results also in the light of the findings about the impact of time-delays and the frequency missmatch on the synchronization of Kuramoto oscillators in brain networks (Petkoski et al PLOS CB 2018), which also hold for other oscillators (Petkoski et al. PTRSA 2019). Similarly, implicit time delays in KM (with phase shifts) were studied by Moon et al Plos CB 2015.

Thanks for introducing these references. We have extended the discussion section to clarify how our study uses and extends the existing knowledge on the communication between brain areas. (please see the subsection “Role of frequency detuning and connection delay”)

- how is the oscillation frequency in Fig 1 defined? Is it a simple ensemble average of frequencies of the firing for all the neurons, or is it the frequency of the locking? For phase oscillators these two can be different in asymmetric networks (as it is the case here with the E/I imbalance), see e.g. Petkoski et al PRE 2013.

We have used the frequency of the populations in the locked state. This is now clearly indicated in Method section and the possible difference is discussed when other definition is used referring to the above reference. (Page 4, line 100)

- would the results be different if there are no time delays within the populations? This approach is for example common when using brain network model, e.g. Sanz Leon Neoroimage 2015.

Indeed internal dynamics of the population have no crucial role in our results once they are in an oscillating state with moderate to high synchrony level. We considered a short time delay for the interaction between neurons within each population to make the model more realistic.

- Can the authors elaborate on the advantage of using populations of neurons instead of neuronal masses such as Wong Wang or Jansen Ritt models? Do they expect at least the PRC and coherence results to be fully confirmed if neuronal masses are used?

Neural mass models have been extensively studied and the role of the PRC in low dimensional oscillator models is well accepted. In this study we intended to show in what extent phase oscillators analysis and the PRC method apply to more realistic and detailed models using populations of spiking neurons.

- the way delta is defined in Eq. 6, requires all the oscillators to be synchronized. Isn’t this too strict condition, and wouldn’t it be more general if the time-delays are explicit in the coupling function, so that the phase shift as defined by the authors still holds, but only for the locked neurons? Similarly, for the simulation of the Kuramoto oscillators, are the the time-delays explicit in the interactions, or there are phase-shifts instead, implying the model to be valid only if all the neurons are synchronized?

We performed simulations with explicit time delays in the Kuramoto oscillators and the results were very similar so we don’t expect that working with explicit time delays will change our main results. Please note that we need to use this approximation for a tractable analytical approach while the simulations with spiking neurons have been done with explicit time delays. We mention that explicit time delays need to be considered only if they are much larger than the period of the oscillations (Izhikevich, *Physical Review E* 58.1 (1998): 905). We added a sentence to clarify this point after Eq. 6. (Page 14, Line 334).

- can it be stated more clearly what is the impact of the assymetry in the coupling, as it was done for example in the above mentioned similar works by Moon et al Plos CB 2015 &Petkoski et al Plos CB 2018?

We added an statement in the first paragraph of the section “Effect of asymmetric connectivity” indicating that the changes in the phase differences due to asymmetric connections, as is shown in Petkoski et al Plos CB 2018, affect the information transmission. (Page 12, Line 304)

- line 395 typo, “In” instead of “If”.

Fixed.

Taken together, I cannot recommend publication of the manuscript in the current form.
